# Health literacy in five districts in Sri Lanka: a baseline assessment of health literacy levels among 18-49-year-olds and associated factors

**DOI:** 10.1186/s12889-025-23641-z

**Published:** 2025-07-15

**Authors:** Millawage Supun Dilara Wijesinghe, Nathasha Hithaishi Obeyesekera, Balangoda Muhamdiramlage Indika Gunawardana, Weerasinghe Mudiyanselage Prasad Chathuranga Weerasinghe, Upeksha Gayani Karawita, Nissanka Achchi Kankanamalage Ayoma Iroshanee Nissanka, Vithanage Chandima Nayani Vithana, Singappuli Arachchilage Sanjeewanie Champika Karunaratne, Praveen Nagendran, Gayani Sandeepika Dissanayake, Ranjith Batuwanthudawe, MAAP Alagiyawanna, Palitha Karunapema

**Affiliations:** 1 Health Promotion Bureau, Colombo, 08 Sri Lanka; 2 Health Information Unit, Ministry of Health, Colombo, 10 Sri Lanka

**Keywords:** Health literacy, Socioeconomic factors, Health communication, Language barriers, Sri Lanka

## Abstract

**Background:**

Despite Sri Lanka’s high general literacy rate, disparities persist in health literacy (HL), which is a critical determinant of healthcare outcomes. This study assessed HL levels among adults aged 18–49 years in five districts and identified the associated sociodemographic and behavioral factors.

**Methods:**

A cross-sectional study was conducted (October 2022 - March 2023) via multistage cluster sampling across five districts (Colombo, Hambantota, Kurunegala, Monaragala, and Mullaitivu). Participants (*n* = 532) were recruited. The validated HLS-EU-Q16 (European Health Literacy Survey- 16-item version) tool was used, and HL was categorized as “limited” (0–12) or “sufficient” (13–16). Multivariable logistic regression was used to analyze the predictors of limited HL. The analysis was conducted via SPSS software (version 23.0).

**Results:**

Overall, 84.6% of the participants demonstrated sufficient HL, whereas 15.4% had limited HL. Regular interaction with public health midwives, the use of television or the internet for health information, and the absence of language barriers significantly reduced the odds of limited HL. Socioeconomic disparities were evident, with 27% lacking access to health information and 17% reporting language-related comprehension challenges.

**Conclusion:**

While Sri Lanka’s primary healthcare infrastructure supports relatively high HL, systemic gaps persist, particularly among linguistically diverse and socioeconomically disadvantaged groups in Sri Lanka. Prioritizing multilingual health communication, digital platforms, and community-based education through frontline health workers can help bridge these gaps. Integrating critical HL competencies into national education and health policies is vital to address the disconnect between general literacy and health empowerment.

## Introduction

Health literacy, defined as the ability to access, understand, appraise, and apply health information, is a cornerstone of effective healthcare decision-making and empowerment [1]. Grounded in Sørensen et al.’s [2] four-dimensional framework (access, understanding, processing, and application) and Nutbeam’s [3] hierarchical model (functional, interactive, and critical literacy), health literacy enables individuals to navigate complex health systems and adopt preventive behavior. Its absence is correlated with adverse outcomes, including poor disease management, inequitable healthcare access and increased morbidity [4].

Globally, health literacy disparities persist, with Southeast Asia exhibiting particularly wide variations (1.6–99.5% prevalence) [5]. Sri Lanka presents a paradox: despite a 92% adult literacy rate [6], emerging data signal alarmingly low health literacy, especially in preventing and managing noncommunicable diseases (NCDs), which account for 71% of the national mortality [7]. This disconnect highlights the urgent need to investigate why high general literacy fails to translate into health competency.

Although valuable, existing Sri Lankan studies focus narrowly on subpopulations, such as school teachers [8] and undergraduates [9]. However, these findings are constrained by nonstandardized methodologies and tools, limiting cross-study comparability and generalizability. Furthermore, the sociodemographic and behavioral determinants of health literacy remain underexplored, hindering the development of targeted interventions [10]. A nationally representative assessment using validated instruments is critical to address these gaps [11]. This study aimed to assess the prevalence of health literacy levels and identify sociodemographic and linguistic determinants among adults aged 18–49 years in five Sri Lankan districts via the validated HLS-EU-Q16 tool.

Enhancing health literacy is pivotal to achieving the World Health Organization (WHO)’s goals of equitable healthcare and noncommunicable disease (NCD) reduction [1]. This study provides actionable evidence for policymakers to design literacy initiatives tailored to vulnerable groups [12, 13], ultimately strengthening public health outcomes in Sri Lanka and similar settings. By harmonizing the methodology with global standards, our findings enable cross-country comparisons and inform context-specific policy reforms.

### Data and methods

**Fig. 1:**
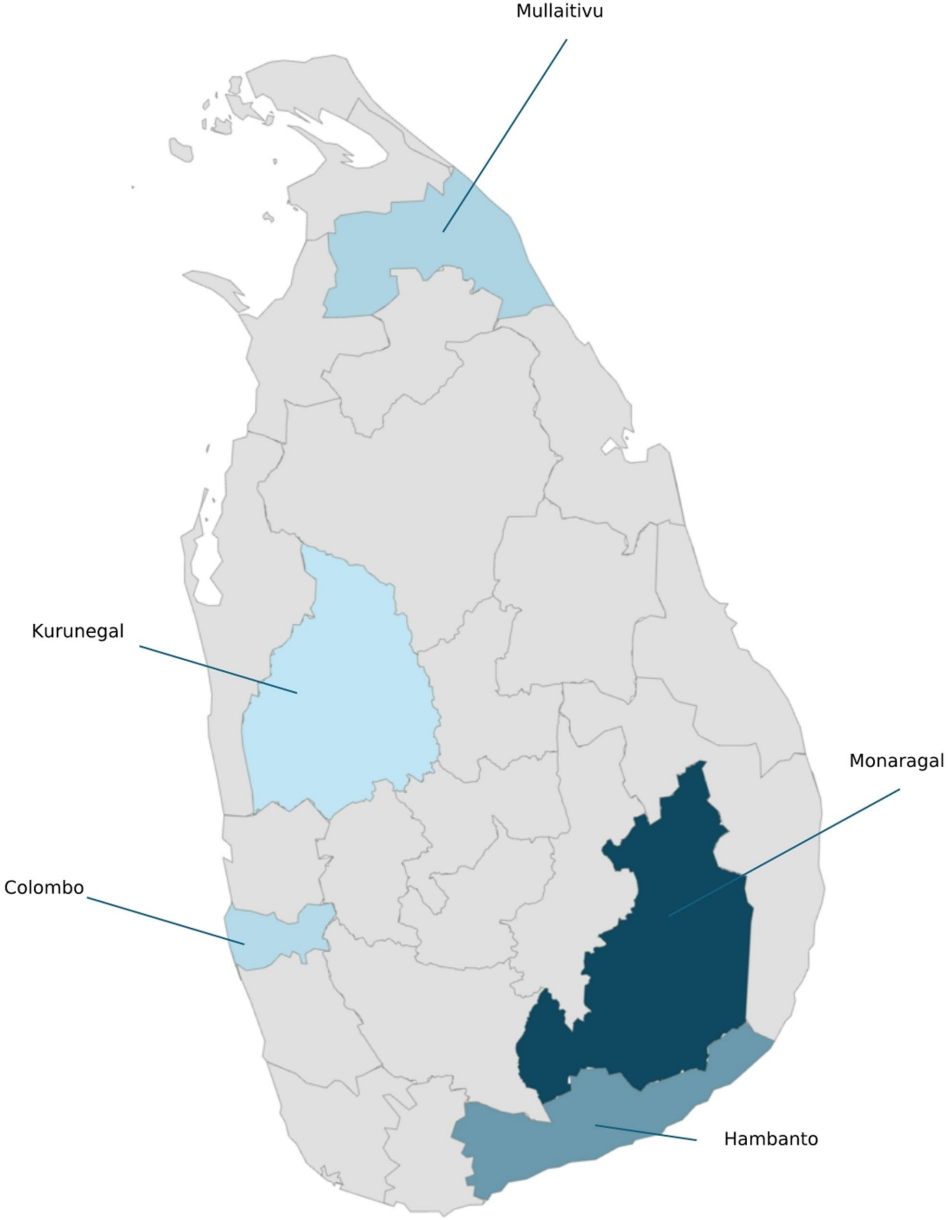
Five districts selected for the study

We conducted a cross-sectional study in five randomly selected districts in Sri Lanka (Fig. 1) from October 1, 2022 to March 31, 2023. The districts were alphabetically ordered and selected via a random number generator to ensure geographic diversity and minimize selection bias. The study population comprised adults aged 18–49 years residing in the selected districts identified through Sri Lanka’s eligible family register, a national public health registry tracking households requiring maternal, child, or family planning services [14]. The 18-49-year-old age group was selected because of its public health significance as Sri Lanka’s working-age population, which is pivotal in household health decisions and NCD prevention. Sampling relied on the eligible family register, a registry tracking households engaged in maternal/child health services, ensuring the representation of adults actively interfacing with primary healthcare. This demographic often manages health choices for dependents, amplifying the impact of their literacy on intergenerational outcomes. Excluding individuals ≥ 50 years of age minimized age-related confounders (e.g., cognitive decline and multimorbidity), thereby enhancing methodological rigor. The selection of this age group balances feasibility, relevance to national priorities, and equity, addressing systemic gaps while leveraging Sri Lanka’s robust primary health care network. Individuals diagnosed with cognitive impairments or psychiatric illnesses were excluded.

The sample size was calculated via the formula of Lwanga and Lemeshow [15] for proportions, where Z = 1.96 (95% confidence level), *p* = 0.5 (maximum variance assumption due to no prior data), and d = 0.05 (margin of error). This yielded an initial sample size of 384. After adjusting for clustering effects (design effect = 2, assuming intraclass correlation ρ = 0.03 [16]) and a 10% nonresponse rate, the final sample size was 853. A three-stage cluster sampling approach with a probability proportional to size was employed: (1) five districts were randomly selected from Sri Lanka’s 26 health districts; (2) 28 clusters (Public Health Midwife (PHM) operational areas [17]) were selected via probability proportional to size sampling on the basis of district population size; and (3) within each cluster, PHMs used a random number table to select one eligible adult per household from the register. The completeness of these registers was assumed to be approximately 100%.

Once the PHM areas were selected in every district, the district focal point of the Health Promotion Bureau and Health Education Officers [18] visited the PHM area and handed over the questionnaires to the PHMs. One eligible adult meeting the inclusion criteria was randomly selected from each family, and the interviewer administered the questionnaire to the participant via the PHM. The tool included two sections: (1) sociodemographic and behavioral factors (age, gender, education, income, marital status, healthcare access, and health information sources) and (2) the validated Sinhala version of the HLS-EU-Q16 tool [19, 20], which categorizes health literacy as inadequate (0–8), problematic (9–12), or sufficient (13–16). It was recategorized as limited (0–12) and sufficient (13–16), where the first two categories (inadequate: 0–8 and problematic: 9–12) were amalgamated to form a single category labeled ‘limited HL’. Permission for the tool’s use was obtained from the local validation team.

Data were analyzed via SPSS 23.0, with descriptive statistics including means (± standard deviations) for continuous variables and proportions for categorical variables. Bivariate analyses revealed associations between sociodemographic factors and health literacy. Variables with *p* < 0.20 in the bivariate analyses were included in a multivariable logistic regression model to assess independent predictors of health literacy, dichotomized as sufficient (13–16) vs. limited (0–12). Adjusted odds ratios (aORs) with 95% confidence intervals (CIs) are reported. Model diagnostics included checks for multicollinearity (variance inflation factor < 5) and goodness-of-fit. Missing data (< 5% across variables) were addressed via complete case analysis, with statistical significance set at *p* < 0.05.

## Results

### Participant characteristics

The study achieved a response rate of 62%, yielding a final sample of 532 adults (mean age = 34.1 years, SD = 7.7 years). The sociodemographic and economic characteristics of the participants are summarized in Table 1. The majority of participants were female (64.1%), of Sinhala ethnicity (86.7%), and married (90.2%). Over 85% reported household incomes below 50,000 LKR/month, and 89.7% were Sinhala speakers. Apart from what is displayed in the table, the majority had no healthcare workers in their families (454, 85.3%). Two hundred and twelve participants (39.8%) met the Medical Officer of Health (MOH), 340 (63.9%) met the PHM, 52 (9.8%) met the Public Health Inspector (PHI), and 26 (4.9%) met other health care workers at least once in the last 6 months. Eighty-nine participants (16.7%) had difficulty understanding an important health-related program or document due to language barriers within the last 6 months. Two hundred ten participants (39.5%) used the internet, 142 (26.7%) used Television (TV as the main source of health information, whereas another 142 (26.7%) did not use any source of health information. One hundred seventy-seven participants (33.3%) believed that expert discussions were the best source of health communication. Only 125 (23.5%) had been members of a health society, and 305 (57.3%) had participated in a health program or competition during their lifetime. One hundred ninety-two participants (36.1%) had a chronic illness themselves or had a family member with a chronic illness.

**Table 1:** Frequency distribution of sociodemographic and socioeconomic factors among participants

**Sociodemographic/socio economic factor**	**Frequency**	**Percent (%)**
District		
Colombo	88	16.5
Hambantota	122	22.9
Kurunegala	144	27.1
Monaragala	127	23.9
Mullaitivu	51	9.6
Gender		
Female	341	64.1
Male	191	35.9
Ethnicity		
Sinhala	461	86.7
Tamil	37	7.0
Muslim	33	6.2
Burgher	1	0.2
Religion		
Buddhist	448	84.2
Hindu	34	6.4
Islam	34	6.4
Christian	16	3.0
Language -Sinhala-speaking		
No	55	10.3
Yes	477	89.7
Language -Tamil-speaking		
No	449	84.4
Yes	83	15.6
Language - English speaking		
No	430	80.8
Yes	102	19.2
Sinhala reading		
No	66	12.4
Yes	466	87.6
Tamil reading		
Yes	97	18.2
No	435	81.8
English reading		
Yes	182	34.2
No	350	65.8
Education level		
No schooling	13	2.4
Up to grade 11 or below	153	28.8
GCE O/L passed	111	20.9
Up to GCE A/L	80	15.0
GCE A/L passed	101	19.0
Diploma, Degree or higher	74	13.9
Marital status		
Single	41	7.7
Married	480	90.2
Widowed	5	0.9
Separated/Divorced	4	0.8
Income level (LKR)		
< 20,000	171	32.1
20,001-30,000	149	28.0
30,001-50,000	134	25.2
50,001-150,000	57	10.7
>150,000	21	3.9

Among the participants, 450 (84.6%) had sufficient health literacy, whereas 68 (12.8%) had problematic health literacy, and 14 (2.6%) had inadequate health literacy.

In the adjusted multivariable analysis examining factors associated with limited health literacy (reference: sufficient health literacy), several significant associations emerged. Participants who regularly met or discussed with public health workers (PHMs) had 42.2% lower odds of having limited health literacy than those who did not (aOR = 0.578, 95% CI: 0.342–0.976, *p* = 0.040). Similarly, using television (aOR = 0.580, 95% CI: 0.343–0.981, *p* = 0.042) or the Internet (aOR = 0.540, 95% CI: 0.326–0.895, *p* = 0.017) as information sources was associated with 42% and 46% reductions in the odds of limited health literacy, respectively. The participants who reported no difficulty understanding health messages due to language barriers had 46.1% lower odds of having limited health literacy than did those who experienced barriers (aOR = 0.539, 95% CI: 0.305–0.953, *p* = 0.034). English-speaking status showed a nonsignificant trend toward lower odds of limited health literacy, despite its significance in the crude analysis. Musculoskeletal diseases were not significantly associated with the outcome, and male sex also lacked significance (Table 2). The multivariable logistic regression model demonstrated significant improvement over the null model, as evidenced by the likelihood ratio test, χ² = 30.5, *p* < 0.001. Model fit indices, including the Akaike information criterion (AIC = 443) and Bayesian information criterion (BIC = 477), indicated reasonable parsimony, given the complexity of the predictors.

**Table 2:** Multivariable analysis of factors associated with limited health literacy

**Predictor**	**Crude odds ratio ** **[95% CI]**	***p*** ** value**	**Adjusted odds ratio ** **[95% CI]**	***p*** ** value**
Sex				
Male	1.489 [0.924-2.401]	0.102	1.147 [0.682-1.927]	0.605
Female (Reference category)				
English speaking				
Yes	0.409 [0.191-0.879]	0.022	0.488 [0.221-1.081]	0.077
No (Reference category)				
Regularly meet or discuss with PHM				
Yes	0.505 [0.314-0.812]	0.005	0.578 [0.342-0.976]	0.040
No (Reference category)				
Use TV as source of information				
Yes	0.580 [0.357-0.941]	0.027	0.580 [0.343-0.981]	0.042
No (Reference category)				
Use internet as source of information				
Yes	0.510 [0.317-0.820]	0.005	0.540 [0.326-0.895]	0.017
No (Reference category)				
Suffering from any form of musculoskeletal diseases				
No	0.356 [0.087-1.450]	0.150	0.379 [0.081-1.775]	0.218
Yes (Reference category)				
Difficulty in understating a health message due to a language barrier at least once (with in last six months)				
No	0.482 [0.279-0.828]	0.008	0.539 [0.305-0.953]	0.034
Yes (Reference category)				

## Discussion

This study provides pivotal insights into health literacy disparities among adults aged 18–49 years in Sri Lanka, where 84.6% demonstrated sufficient HL and 15.4% faced limitations. These findings align with global HL variability in Southeast Asia, where reported adequacy ranges from 1.6 to 99.5% [5], influenced by methodological heterogeneity and sociocultural contexts [21]. However, Sri Lanka’s HL prevalence does not diverge sharply from that of regional countries such as India (72.6% of adolescents had moderate HL) [22] and Indonesia (74% of university staff had adequate HL) [23]. Although the values are similar, it is important to note that in our study, we assessed literacy among the general population aged 18–49 years, compared with other studies that assessed adolescent and young adult age groups. This may reflect Sri Lanka’s robust primary healthcare infrastructure and near-universal education access [24] but emphasize persistent inequities in translating basic literacy into health-related competencies.

A critical paradox emerges between Sri Lanka’s 92% general adult literacy rate [24] and the persistence of limited HL among 15% of the respondents. This aligns with global evidence that functional literacy focused on reading and writing often fails to equip individuals with the skills to critically evaluate or apply health information, particularly in fragmented health systems [25, 26]. Nutbeam’s hierarchical framework [25] distinguishes functional HL from interactive and critical HL, which require advanced skills such as navigating digital platforms or interpreting chronic disease risk. Sri Lanka’s education system, while achieving high enrollment rates, may inadequately prioritize these higher-order competencies, mirroring the challenges observed in Thailand and Malaysia, where the curricula lack HL integration [27–29]. Systemic inequities further exacerbate these gaps; 27% of participants reported no engagement with health information sources, reflecting barriers similar to those in rural India and Indonesia, where socioeconomic disparities limit access [30, 31]. Additionally, the language barriers reported by 17% of the respondents emphasize the urgency for linguistically inclusive health communication in Sri Lanka’s multilingual society, a priority emphasized by UNESCO [32] in Lower- and Middle- Income Countries (LMIC) contexts.

The findings of this study align closely with the international literature, highlighting the critical role of regular interactions with primary healthcare professionals in enhancing health literacy and improving health outcomes. Consistent with previous studies, regular meetings or discussions with primary healthcare providers were associated with improved comprehension of health messages, echoing the findings reported by Nutbeam [3], who emphasized that continuous patient–provider communication is vital for patient education and engagement. Our findings also support international evidence regarding the role of mass media, particularly television, in positively influencing public health message comprehension, similar to the findings of Wakefield et al. [33] on the effectiveness of television campaigns in public health interventions. Additionally, the identified benefit of using the internet for health information aligns with studies highlighting the growing importance of digital platforms in promoting health literacy [34], although contrasting findings suggest variability in internet efficacy on the basis of demographic factors, such as age and socioeconomic status [35]. Finally, our results emphasize that language barriers significantly impact health message comprehension, reinforcing international evidence that overcoming linguistic barriers is crucial for equitable healthcare access and improved health outcomes [36], although the magnitude of these effects can vary by region and cultural context.

Methodologically, while the use of the validated HLS-EU-Q16 tool [19, 20, 37] strengthens reliability, the cross-sectional design limits causal inferences between HL and sociodemographic factors in this study. Self-reported data, although pragmatic, may introduce social desirability bias, a recurring limitation in LMIC studies [38]. The 62% response rate was comparable to that of surveys in resource-limited settings [39, 40]. Although a low response rate may decrease generalizability and the effect of sample size, representativeness was ensured through random cluster sampling and demographic diversity. Furthermore, excluding adults aged ≥ 50 years overlooks a demographic increasingly vulnerable to chronic diseases, and the prevalence of HL has declined, as evidenced by aging populations globally [41].

These findings necessitate the integration of HL into Sri Lanka’s national health and education agendas through three key strategies. First, curriculum reforms should embed critical HL competencies in evaluating misinformation and understanding preventive care into secondary and tertiary education, guided by the WHO’s health-promoting school framework [42]. Community-based programs led by frontline health workers, such as mothers’ support groups [43], can bridge adult HL gaps through door-to-door education. Second, multilingual campaigns codesigned with local communities are imperative. Furthermore, digital platforms such as WhatsApp, which have been adapted for health messaging, could be tailored for Tamil- and Sinhala-speaking populations [44, 45]. Third, health systems must adopt HL-sensitive communication (easy to understand, culturally appropriate, and accessible communication), train providers in plain language and visual aids (training healthcare providers in verbal and written communication and embedding health literacy training in undergraduate and graduate education), and reduce disparities through the use of patient navigation tools [46].

Future research should prioritize longitudinal designs to disentangle the causal relationship between HL and health outcomes, particularly for Sri Lanka’s increasing chronic disease burden. Mixed-methods studies could explore sociocultural barriers, such as stigma or reliance on traditional medicine, which quantitative tools may overlook. Regionally, harmonizing HL metrics via tools such as the HLS [37] would enable cross-border benchmarking, fostering collaborative interventions across South Asia. Inclusive research encompassing older adults, rural communities, and ethnic minorities is critical to ensure equitable advances in HL.

The findings of this study should be interpreted considering several limitations. Because the research employed a cross-sectional design, it offers only a snapshot in time; neither temporality nor causality can be established. All the measurements relied on self-reported information; therefore, social desirability bias may have led the participants to overestimate their health literacy levels. Although random cluster sampling was used, the 62% response rate and noninclusion of adults aged 50 years raised the possibility of affecting the generalizability of the findings. Finally, because the data were gathered only in selected areas rather than across all 26 health districts in Sri Lanka, the results may not be fully generalizable to the entire country.

## Conclusions

In conclusion, this study not only maps HL disparities in Sri Lanka but also highlights the systemic gaps between functional literacy and health ability. By anchoring equity-focused multisectoral strategies spanning education, digital innovation, and healthcare, Sri Lanka can model HL advancement for LMICs in light of similar challenges. This further emphasizes the importance of embedding health literacy into national health policies and educational reforms. Communication between healthcare workers and the public must occur through culturally and linguistically appropriate platforms.

## Data Availability

The datasets used in this study are available from the corresponding author upon reasonable request.

## Abbreviations

HL: Health Literacy
HLS-EU-Q16: European Health Literacy Survey-, 16-item version
SPSS: Statistical Package for the Social Sciences
NCDs: Non-Communicable Diseases
WHO: World Health Organization
CI: Confidence Interval
PHM: Public Health Midwife
aOR: adjusted Odds Ratio
SD: Standard Deviation
LKR: Sri Lankan Rupees
MOH: Medical Officer of Health
PHI: Public Health Inspector
TV: Television
GCE O/L: General Certificate of Education Ordinary Level
GCE A/L: General Certificate of Education Advanced Level
χ²: Chi-squared
AIC: Akaike Information Criterion
BIC: Bayesian Information Criterion
UNESCO: United Nations Educational, Scientific and Cultural Organization
LMIC: Low-and Middle-Income Countries
